# Drive for muscularity behaviors in male bodybuilders: a trans-contextual model of motivation

**DOI:** 10.1186/s40337-019-0274-y

**Published:** 2019-12-31

**Authors:** Lisa Chaba, Fabienne d’Arripe-Longueville, Vanessa Lentillon-Kaestner, Stéphanie Scoffier-Mériaux

**Affiliations:** 1 University of Teacher Education of the State of Vaud, (HEP-VD), Avenue de Cour 25, 1014 Lausanne, Switzerland; 2 Université Côte d’Azur, LAMHESS, Nice, France

**Keywords:** Drive for muscularity behaviors, Male bodybuilders, Trans-contextual model of motivation, Self-determination theory, Theory of planned behavior

## Abstract

**Background:**

The drive for muscularity behaviors are very common in male athletes, especially in male bodybuilders. Studies have related drive for muscularity behaviors to body dissatisfaction, eating disorders and muscle dysmorphia.

**Methods:**

This study applied the trans-contextual model of motivation to the drive for muscularity behaviors of male bodybuilders at risk of developing muscle dysmorphia. The relationships between self-determination theory constructs and drive for muscularity behaviors, via the theory of planned behavior variables (i.e., attitude, subjective norm, perceived behavioral control, and intention) were examined. A total of 175 Swiss male bodybuilders (*M*_age_ = 27.34; *SD*_age_ = 7.53) completed measures on motivation for sport, theory of planned behavior variables, and drive for muscularity behaviors. They practiced bodybuilding from three to 24 h per week (*M*hours per week = 6.59; *SD*hours per week = 3.45) and had done so for 7.19 years on average (*SD*number of years = 6.91). Using bootstrapped maximum likelihood estimation with the AMOS 7.0 program, a series of confirmatory factor analyses was performed on each subscale and a series of path analyses was performed to determine the final model.

**Results:**

The fit indices of the final model were satisfactory: χ^2^ (11) = 13.81; *p* = .244; TLI = .98; CFI = .99; RMSEA = .04. The model explained 29% of the variance of drive for muscularity behaviors. The final path analysis supported the motivational sequence, with autonomous motivation for sport showing a positive, significant and indirect association with the drive for muscularity behaviors via perceived behavioral control and intention to gain muscle mass, and controlled motivation for sport showing a positive association with the drive for muscularity behaviors both directly and via attitude and intention to gain muscle mass.

**Conclusions:**

It was concluded that the trans-contextual model of motivation applies only partially to the drive for muscularity behaviors in male bodybuilders.

**Perspective:**

The motivational mechanisms explaining the development of drive for muscularity behaviors might be better understood through complementary analyses of motivational profiles. Such investigations would guide the design of programs to lower the risks associated with these behaviors.

## Plain English summary

The strengths of the study include the application of the trans-contextual model of motivation to the drive for muscularity behaviors of male bodybuilders at risk of developing muscle dysmorphia. In this model, motivations for sport influence drive for muscularity behaviors through the mediating roles of attitude to gain muscle mass, perceived behavioral control related to gain muscle mass, and intention to gain muscle mass. The findings were that both autonomous and controlled motivations for sport were associated with drive for muscularity behaviors through direct and indirect pathways. Complementary analyses of motivational profiles would provide more in-depth insight into the motivational mechanisms underlying drive for muscularity behaviors. These investigations would help to develop programs to reduce the risks associated with these behaviors.

## Background

Athletes practicing bodybuilding display drive for muscularity behaviors (DM) and are at risk of developing deviant behaviors that can impair health [1], a notable example being the development of eating disorders [2]. Although several psychological factors have been shown to be related to DM, few studies have been based on recent socio-cognitive theories. The purpose of this study was to gain deeper insight into the psychological mechanisms underlying DM in male bodybuilders from the perspective of the trans-contextual model of motivation (TCM) of Hagger and Chatzisarantis (2009) [3].

### Drive for muscularity behaviors (DM)

Bodybuilding is an extreme sport practice in which participants train to build substantial muscle volume while maintaining muscle definition [2, 4], but it is also a life style for athletes who want to develop a physique with ideal proportions [5]. Although, bodybuilding may be an alternative response to disordered body image in men [6, 7], certain fitness activities, including bodybuilding, have been deemed risk factors for the development of body dissatisfaction [8], obsessive mental disorders like muscle dysmorphia [9, 10] and eating disorders [11, 12].

Drive for muscularity was the term coined by McCreary and Sasse (2000) [9] to describe an individual’s motivation to become more muscular. Several studies [10] have shown that in the bodybuilding context, DM is associated with significant dietary restraint, with both sugar ingestion and caloric intake are greatly reduced and protein intake is greatly increased [2, 13]. This eating plan can sometimes lead to binge eating behaviors [14] and generally heighten the risk of developing an eating disorder [15, 16]. High levels of DM have been associated with several sociodemographic variables, such as adolescence (i.e., 16–17 years old), sex orientation (i.e., gay men), sport category (e.g., weight sports, aesthetic sports), and sport level (competitors) [1, 17]. As men living in Western countries are more likely to focus on improving their muscle mass, they tend to become more involved in bodybuilding and are therefore at greater risk of DM [17, 18]. Numerous personality traits such as emotional dysregulation and perfectionism [14], anxiety [19], and depression [20] have been related to DM, whereas self-esteem seems negatively related to this variable [14, 19]. Higher levels of DM might be associated with negative outcomes like poor self-esteem and higher levels of depression [9]. Societal norms and social influences have also been shown to be involved in the desire to increase muscle mass [21].

Some studies have investigated DM through the lens of self-determination theory. Edwards et al. (2016) [22] showed that autonomy moderates the relation to DM and the internalization of the mesomorphic ideal. Selvi and Bozo [23] underlined that DM moderates the association between frustrated basic needs and muscle dysmorphia. These previous studies did not use the self-determination continuum, but they encouraged future studies to do so. In addition, the roles of socio-cognitive variables like attitude, subjective norm and perceived behavioral control, need to be more fully explored in relation to DM.

### The trans-contextual model of motivation (TCM)

Researchers have recently advocated integrated approaches to gain more comprehensive explanations of the factors and mechanisms that influence health behaviors. The TCM from Hagger and Chatzisarantis (2009) [3], which combines self-determination theory [24] and the theory of planned behavior [25], has been applied in various contexts, mainly physical activity and physical education [26–28] and academic settings [29, 30]. Other studies have focused on health-related behaviors like healthy eating [31, 32] and doping intention [33]. This model might offer a heuristic theoretical framework to better understand DM and the risk of eating disorders in male bodybuilders.

#### Self-determination theory [24]

Self-determination theory distinguishes self-determined (autonomous) from non-self-determined (controlled) forms of motivation along a continuum of perceived locus of causality (PLOC, [34]. Autonomous motivation is intrinsic; it lies at one extreme of the PLOC continuum and reflects acting to satisfy personally relevant goals. Identified regulation is also an autonomous form of motivation and refers to motivation to engage in behavior because it serves internally referenced and highly valued goals. Controlled motivation is extrinsic; it lies at the opposite extreme of the continuum and reflects engaging in behaviors because of external reinforcement. Introjected regulation is also a controlled form of motivation and reflects behavioral engagement due to perceived internal pressures.

#### The theory of planned behavior [25]

In this theory, intention is assumed to mediate the effects of attitude, subjective norm, and perceived behavioral control on actual behavior. Attitude defines the general belief that the target behavior will result in certain desirable outcomes; subjective norm reflects the belief that significant others desire the individual to perform the target behavior; and perceived behavioral control represents the belief that the individual has the capacities, faculties, abilities, and resources to engage in the target behavior. Perceived behavioral control is also hypothesized to have a direct effect on actual behavior [35].

#### The trans-contextual model of motivation (TCM) [3]

**Fig. 1:**
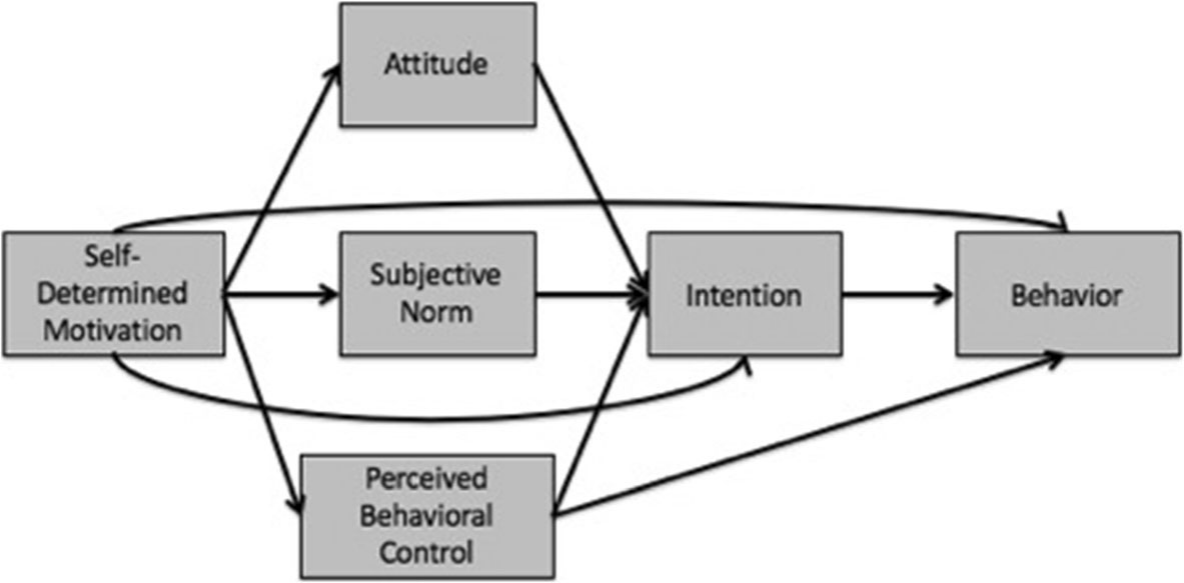
The TCM of Hagger and Chatzisarantis (2009) [3]

Recently, an integrated approach that incorporates self-determination theory and the theory of planned behavior was shown to be effective (see Fig. 1). This approach was based on the idea that self-determination theory would provide information about the origins of the socio-cognitive variables from the theory of planned behavior that influence behavior. Similarly, it was assumed that the socio-cognitive variables from the theory of planned behavior would delineate the mechanisms by which the motivational constructs from self-determination theory influence behavior.

A growing body of literature has examined various applications of the trans-contextual model to healthy behaviors such as healthy eating [31], and physical activity [3, 36, 37], or to unhealthy behaviors like doping [33]. As DM is a risk factor for developing deviant behaviors, autonomous motivation, which is known to be negatively related to eating disorders and doping use could also be expected to be negatively related to DM [33]. However, people who strive for muscularity might be quite intrinsically motivated toward this goal. Applying the TCM to the context of DM in male bodybuilders might provide deeper insight into the psychological mechanisms underlying the risk of developing deviant behaviors in this population. Furthermore, the findings of this study would add to the literature by contributing evidence of the generalizability of the model to multiple health behavior domains.

### Aims of the study

**Fig. 2:**
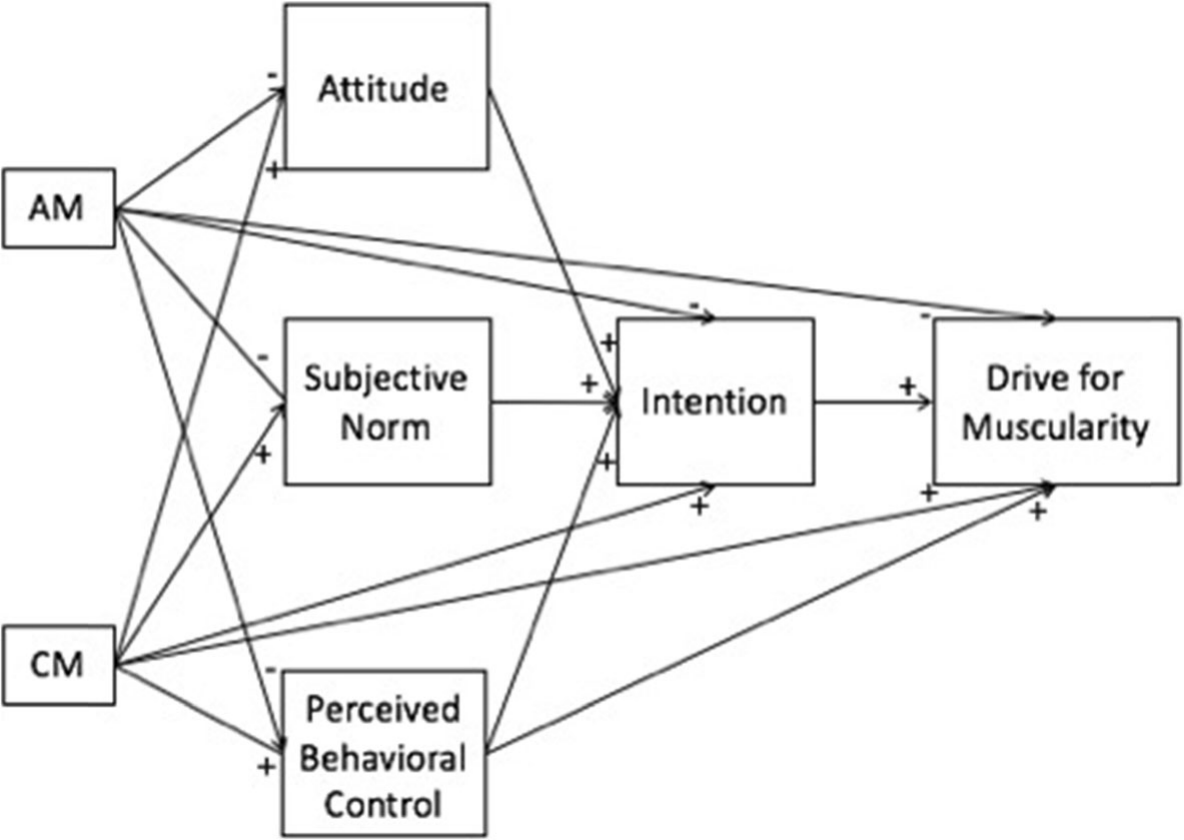
Hypothetical application of Hagger and Chatzisarantis’s (2009) [3] TCM to DM in male bodybuilders. *Note.* AM = autonomous motivation for sport; CM = controlled motivation for sport; Attitude = attitude to gain muscle mass; Subjective norm = subjective norm related to gain muscle mass; Perceived behavioral control = perceived behavioral control related to gain muscle mass; Intention = intention to gain muscle mass

The purpose of this study was to test the application of Hagger and Chatzisarantis’s (2009) TCM [3] to DM in male bodybuilders. The model was developed to be generalizable across contexts and populations, and the theories on which the model is based adopt a similar perspective [38]. Therefore, based on the tenets of this model [3] and related research, we tested a hypothetical model (see Fig. 2) and the following hypothesized pathways:

1. Autonomous and controlled motivations for sport are, respectively, negative and positive predictors of the socio-cognitive variables from the theory of planned behavior (i.e., attitude, subjective norm, perceived behavioral control, and intention to gain muscle mass).
2. Autonomous and controlled motivations for sport are, respectively, negative and positive predictors of DM.
3. The relationships between motivations for sport and DM are mediated by the socio-cognitive variables from the theory of planned behavior.
4. The indirect associations between motivations for sport and DM are stronger than the direct associations.

## Methods

### Participants

This study was conducted with 175 French-speaking Swiss male athletes practicing bodybuilding who met the following eligibility criteria: (a) minimum age of 16 years, (b) minimum of 3 h of physical training per week, and (c) minimum of 3 years of bodybuilding. Participants were 17–57 years old (*M*age = 27.34; *SD*age = 7.53), practiced the sport from 3 to 24 h per week (*M*hours per week = 6.59; *SD*hours per week = 3.45), and had been bodybuilders for 7.19 years on average (*SD*number of years = 6.91).

### Procedure

The ethics committees of the University of Teacher Education of the State of Vaud (Switzerland) and the University of Nice Sophia-Antipolis (France) approved the protocol design and the study. Data were collected over 6 months and participants were recruited in a social network or in gyms. Written informed consent was obtained from the participants prior to their participation (or their parents in the case of minors). Online survey completion did not exceed more than 20 min and responses to all questions were obligatory; there were no missing data. Participants were informed beforehand that the survey was not a test (i.e., there were no right or wrong answers) and that all responses would be used for research purposes only. Participation was entirely voluntary and full confidentiality was guaranteed. Authors collected information about nationality in order to ensure that participants were French speakers.

### Measures

In this section, Cronbach’s alphas higher than .70 were considered satisfactory and those between .60 and .69 were considered “marginally acceptable“ for all measures, in line with the recommendations of Briggs-Gowan and Carter (1998) [39].

### Sport motivation

Sport motivation was measured using 19 items from the French version of the Behavioral Regulation in Exercise Questionnaire (BREQ-2 [40];). Participants answered each item with a Likert scale ranging from 1 (not at all) to 6 (absolutely). They were asked to rate several reasons pertaining to four regulation styles varying in the degree of autonomy on a continuum ranging from high to low autonomy: extrinsic regulation, introjected regulation, identified regulation, and intrinsic regulation. Extrinsic regulation is the least autonomous form of motivation, while intrinsic motivation is the most autonomous. Autonomous motivation was represented by eight items corresponding to intrinsic regulation (four items; e.g., *I do sports because I think exercise is fun)* and identified regulation (four items; e.g., *I do sports because I value the benefits of exercise*). Controlled motivation was represented by seven items corresponding to extrinsic regulation (four items; e.g., *I do sports because other people say I should*) and introjected regulation (three items; e.g., *I do sports because I feel guilty when I don’t exercise*). The BREQ-2 has been shown to have good psychometric properties [40]. In the present study, the internal reliabilities of the autonomous motivation and controlled motivation subscales were respectively acceptable (α_AM_ = .72) and marginally acceptable (α_CM_ = .67) [39].

### Theory of planned behavior constructs

The items related to participants’ beliefs about DM (i.e., constructs of attitude, subjective norm, perceived behavioral control, and intention to gain muscle mass) were developed and adapted on the basis of previous work on the theory of planned behavior [25, 41]. A 6-point Likert scale from 1 (not at all) to 6 (absolutely) was used. For each scale, the CFA was computed and Cronbach’s alphas were performed to verify the internal consistency of each construct.

### Attitude

Four items related to the perceived benefits of gaining muscle mass were used (e.g., *I think I would be more self-confident if I had more muscle mass*). The CFA provided a good fit to the data: χ^2^ (5) = 5.9; *p* = .311; TLI = .99; CFI = .99; RMSEA = .02. The internal consistency of this subscale was satisfactory (αAttitude = .77).

### Subjective norm

Subjective norm related to gaining muscle mass was measured through four items (e.g., *My peers approve of me trying to increase my muscle mass*). The CFA provided a good fit to the data: χ^2^ (2) = 3.6; *p* = .169; TLI = .97; CFI = .99; RMSEA = .06. The internal consistency of this subscale was satisfactory (αSubjective norm = .74).

### Perceived behavioral control

Five items related to perceived behavioral control related to gaining muscle mass were used (e.g., *I feel able to do intensive strength training*). The CFA provided a good fit to the data: χ^2^ (7) = 31.6; *p* = .059; TLI = .95; CFI = .97; RMSEA = .07. A satisfactory Cronbach’s alpha value of .76 was obtained for this subscale.

### Intention

The measure of the intention to gain muscle mass was composed of three items (e.g., *I intend to gain muscle mass*). The CFA provided a good fit to the data: χ^2^ (1) = 1.9; *p* = .158; TLI = .98; CFI = .99; RMSEA = .07. The internal consistency of the subscale was satisfactory (α_Intention_ = .77).

### Drive for muscularity behaviors

The Drive for Muscularity Scale (DMS [9];) was initially composed of two subscales: “attitudes“ and “behaviors.“ Although the DMS is the scale most often used, several limitations have been noted, such as the lack of theoretical validity and the lack of differentiation between attitudes of DM and the behaviors related to DM [42, 43]. The last version of the DMS, validated in French (DMS-FR [44];), is composed of two new subscales: “muscularity body dissatisfaction“ and “muscularity behaviors“. The five items of the Muscularity Behaviors subscale (MB; e.g., *I lift weights to build up muscle*) of the French version of the Drive for Muscularity Scale **(**DMS-FR [44];) were used. The items were answered with a Likert scale from 1 (not at all) to 6 (absolutely). The internal reliability of this subscale was marginally acceptable (α_MB_ = .67) [39].

### Data analysis

This study included several types of analysis. First, confirmatory factor analyses (CFA) were performed to verify the validity of the scales. Second, a series of path analyses was performed to test the hypothetical model. The CFA series used bootstrapped maximum likelihood estimation with the AMOS 7.0 program [45]. The CFA of each subscale was examined with relative fit indices as recommended by Hu and Bentler (1999) [46] because the goodness-of-fit chi-square that compares the hypothesized model with the independent or “totally free“ model is almost always significant, even for well-fitting models, making it an inadequate basis for model evaluation. Therefore, the Comparative Fit Index (CFI), the Tucker-Lewis Index (TLI), and the Root Mean Square Error of Approximation (RMSEA) were used to evaluate model fit because simulation studies have shown that these fit indices provide relatively consistent and stable assessments [47]. A cutoff value of .90 or above for the TLI and CFI is typically considered an acceptable criterion for model fit, although a value greater than .95 is preferable [46]. A critical value of .08 or below for the RMSEA was considered satisfactory for good fit [46]. The model was rejected if the probability value (*p*) was below .05 [48]. Modification indices were used to flag fixed parameters in the model that would make a significant change in the goodness-of-fit chi-square value if freed, and the likelihood-ratio test based on the goodness-of-fit chi-square was used to identify misspecifications in the constrained models from the invariance analyses relative to the baseline model.

The series of path analyses was performed using bootstrapped maximum likelihood estimation with the AMOS 7.0 program [45]. Seven factors were incorporated: autonomous motivation, controlled motivation, attitude, subjective norm, perceived behavioral control, intention to gain muscle mass, and DM. In order to define the scale of the factors and to ensure that the model was properly identified, one indicator for each factor was arbitrarily set to the value of one. In addition, all the latent factors were freely correlated, as is the norm in path analysis. Non-significant links were removed in accordance with the recommendations of MacCallum (1986) [49]. As previously presented, assessment of model fit was based on multiple indicators [46, 50]: χ^2^, CFI, TLI and RMSEA [47]. The analyses were validated according to the same criteria as the previous analyses [46, 50]. Because χ^2^ difference tests cannot be legitimately performed on non-nested models, Akaike’s information criterion (AIC) and the expected cross validation index (ECVI) were used. The AIC value was computed based on the chi-square value for the model minus two times the number of estimated parameters [51]. The ECVI is a single sample estimate that indicates how well the current solution fits in an independently drawn sample [52]. The AIC and ECVI were not normed on a zero to one scale. Reductions in their values, in comparison with other competing models, demonstrated an improved and more parsimonious fit of a model [53].

The direct and indirect effects (i.e., comprising all the direct paths and all the indirect paths from one variable to another) and the total effects (i.e., comprising all the direct paths and all the indirect paths) for the structural model were calculated [54]. Methods of multiple mediation were adopted, and the different effects and their corresponding 95% CIs were calculated to estimate both total and indirect effects for the multiple mediator models, using bootstrapping and providing bias-corrected 95% CIs. The number of bootstrap draws specified was 10,000 as recommended by Hayes (2012) [55].

The series of path analysis tests began with the first model that incorporated the seven factors (i.e., autonomous motivation, controlled motivation, attitude, subjective norm, perceived behavioral control, intention to gain muscle mass, and DM) and all the links were tested as the hypothetical model. The second model also incorporated the seven factors but all non-significant links observed in the previous model were removed, as recommended MacCallum (1986) [49]: the direct links from autonomous motivation to attitude, subjective norm, and DM; from controlled motivation to perceived behavioral control; from subjective norm to intention to gain muscle mass; from controlled motivation to intention to gain muscle mass; and from perceived behavioral control to DM. The third model was also composed of the seven factors and all relationships that were significant in the previous model. The attitude error and the perceived behavioral control error were related to have a better fit index for the final model. RMSEA, TLI, and CFI were acceptable, and the probability value (*p*) was below .05.

## Results

Descriptive statistics of the variables, reliability coefficients, and a Pearson correlation matrix of the major variables are presented in Table 1.

**Table 1:** Descriptive statistics, reliability coefficients, and Pearson correlations (*N* = 171)

Variables	Mean (SD)	1	2	3	4	5	6	7
1. Autonomous motivation for sport	6.22 (0.66)							
2. Controlled motivation for sport	2.21 (0.77)	.72						
3. Attitude to gain muscle mass	4.31 (1.57)	-.07	.37**					
4. Subjective norm related to gain muscle mass	3.29 (1.18)	.01	.18*	.34**				
5. Perceived behavioral control related to gain muscle mass	5.92 (1.07)	.34**	.02	.21**	.16*			
6. Intention	5.09 (1.64)	.29**	.23**	.49**	.28**	.56**		
7. Drive for muscularity behaviors	3.25 (1.42)	.21**	.34**	.33**	.14	.31**	.50**	

* *p* < .05; ** *p* < .01. The Cronbach alpha values are reported on the diagonal of the matrix in the table

**Fig. 3:**
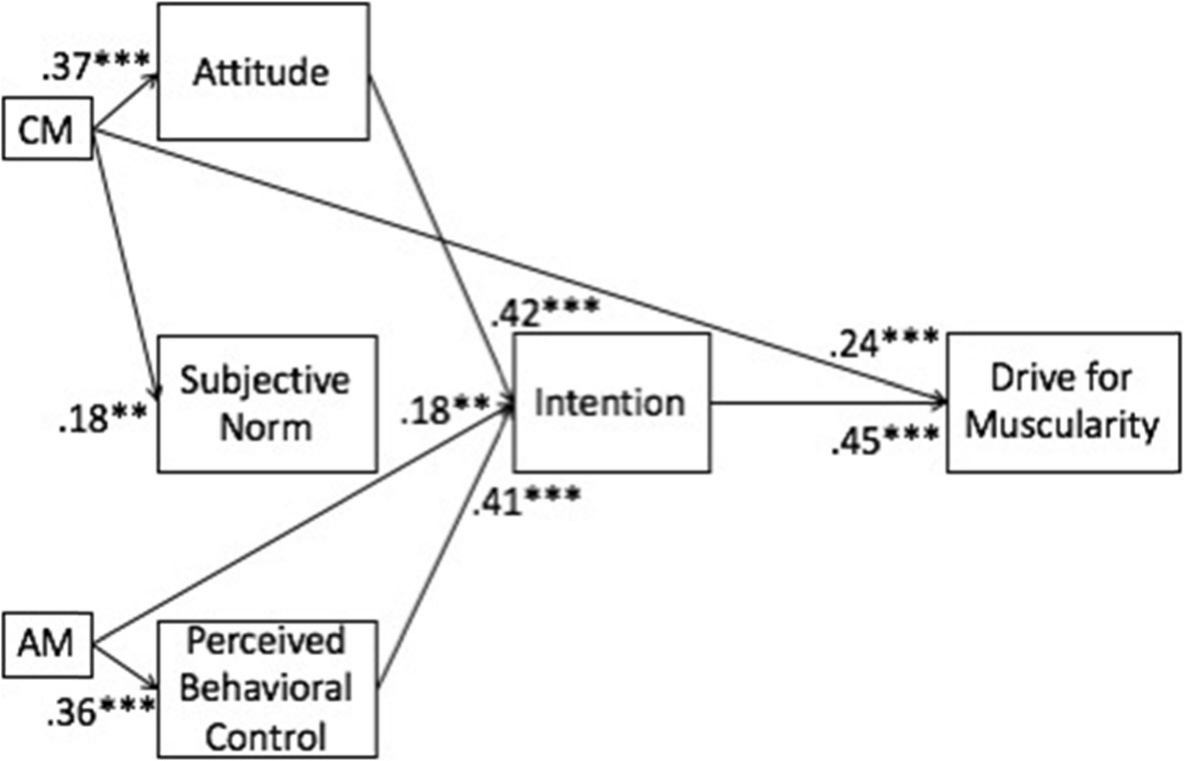
Final path analysis of the TCM applied to DM among male bodybuilders *Notes.* CM = controlled motivation for sport; AM = autonomous motivation for sport; Attitude = attitude to gain muscle mass; Subjective Norm = subjective norm related to gain muscle mass; Perceived Behavioral Control = perceived behavioral control related to gain muscle mass; Intention = intention to gain muscle mass. * *p* < .05; ** *p* < .01; *** *p* < .001

To test the hypothesized relationships between the variables, the series of path analyses was performed and is presented in Table 2, showing that the goodness-of-fit indices were acceptable. The final model was model 3, which demonstrated the best goodness-of-fit indices, as well as the lowest ECVI and AIC indices: χ^2^ (11) = 13.81; *p* = .244; TLI = .98; CFI = .99; RMSEA = .04. The structural path coefficients are shown in Fig. 3. The model explained 29% of the variance of DM.

**Table 2:** Series of path analyses for the final structural model

	χ^2^ (df)	*p*	RMSEA	TLI	CFI	AIC	ECVI
Model 1	33.1 (6)	.000	.16	.62	.89	91.1	.524
Model 2	39.6 (13)	.000	.11	.83	.89	83.6	.480
Model 3	13.8 (11)	.244	.03	.98	.99	61.8	.355

χ^2^ = chi square; *RMSEA* Root mean square error of approximation, *TLI* Tucker-Lewis index, *CFI* Comparative fit index, *AIC* Akaike’s information criterion, *ECVI* Expected cross validation index

Autonomous motivation for sport was significantly, directly and positively related to the intention to gain muscle mass (*ß* = .18, *p* < .01), but non-significantly related to DM. Autonomous motivation for sport was significantly, indirectly and positively related to DM through the mediating role of perceived behavioral control (*ß* = .36, *p* < .001) and the intention to gain muscle mass (*ß* = .41, *p* < .001). No significant relationships between autonomous motivation for sport and the other theory of planned behavior variables (i.e., attitude to gain muscle mass and subjective norm related to gain muscle mass) were found. Controlled motivation for sport was significantly, directly and positively related to DM (*ß* = .24, *p* < .001). Moreover, controlled motivation for sport was also significantly, indirectly and positively related to DM through the mediating role of attitude (*ß* = .37, *p* < .001) and the intention to gain muscle mass (*ß* = .42, *p* < .001). Controlled motivation for sport was significantly and positively related to subjective norm related to gain muscle mass, well (*ß* = .18, *p* < .01), but this variable was non-significantly related to the intention to gain muscle mass.

To further examine the mediating role of the theory of planned behavior variables in the relationships between motivations for sport and DM, we performed multiple mediation analyses following the recommendations of Hayes (2012) [55]. The results are presented in Table 3 and showed that autonomous motivation for sport was significantly, indirectly and positively related to DM through perceived behavioral control and intention to gain mass (i.e., *ß*_AM-DM_ = .45, *p* < .001), while controlled motivation for sport was positively related to DM through attitude and intention to gain muscle mass (*ß*_CM-DM_ = .62, *p* < .001). Last, these indirect associations between motivations for sport and DM were positive and stronger than the direct associations (i.e., *ß*_AM-DM_ = 13, *p* = .403; *ß*_CM-DM_ = .41, *p* < .01).

**Table 3:** Summary of multiple mediation analyses for the final structural model

Independent variable	First mediator variable	Second mediator variable	Dependent variable	*a* path coef	*b* path coef	*c* path coef	*c’* path coef	*d* path coef	Mean indirect effect	SE of mean	Bias-corrected 95% CI mean effect
AM	PBC	INT	DM	.56***	.40***	.45**	.13	.80***	.18	.05	[.09 to .30]
CM	Att	INT	DM	.75***	.37***	.62***	.41**	.50***	.14	.04	[.07 to .24]

*AM* autonomous motivation for sport, *CM* controlled motivation for sport, *PBC* perceived behavioral control related to drive for muscularity, *Att* attitude related to drive for muscularity, *INT* intention to gain muscle mass, *DM* drive for muscularity, *a* direct effect of the independent variable on the first mediator variable, *b* direct effect of the second mediator variable on the dependent variable, *c* indirect effect of the independent variable on the dependent variable through mediator variables, *c’* direct effect of the independent variable on the dependent variable, *d* direct effect of the first mediator variable on the second variable, *95% CI* lower and upper bounds of bias-corrected 95% confidence interval with 10,000 bootstrap samples

** *p* < .01; *** *p* < .001

## Discussion

The aim of the present study was to apply the key propositions and hypotheses of the TCM [3] to DM in male bodybuilders. The results partially supported the tenets of the TCM, thus enriching our theoretical understanding of how motivational dynamics operate on specific behaviors in sport. We hypothesized that motivations for sport would affect DM both directly and via the mediation of socio-cognitive variables from the theory of planned behavior.

Autonomous and controlled motivations for sport were expected to be, respectively, negative and positive predictors of the socio-cognitive variables from the theory of planned behavior (i.e., attitude, subjective norm, perceived behavioral control, and intention to gain muscle mass). However, the results showed that autonomous motivation for sport was significantly related only to perceived behavioral control and intention to gain muscle mass, and these relationships were positive. Such positive associations have been reported in previous research that showed autonomous motivation to be a protective factor for positive health behaviors such as doping avoidance [33], healthy eating [31], and physical activity [3, 36, 37]. Our finding indicates that the more self-determined male bodybuilders are in sport, the more they perceive they can control their behaviors related to gain muscle mass and had the intention to gain muscle mass. This suggests that autonomous motivation is related to any behavior that requires effort, whether that behavior is healthy or unhealthy, and whether the population wants to lose weight and maintaining a diet [56] or wants to gain muscle mass.

Controlled motivation for sport was expected to be a positive predictor of each of the socio-cognitive variables from the theory of planned behavior. In accordance with previous studies, we found that controlled motivation for sport was significantly and positively related to attitude [32] and subjective norm [33, 38, 57], but not to perceived behavioral control [58]. Moreover, and in accordance with previous studies [27, 29], subjective norm related to gain muscle mass was the only variable non-significantly related to intention to gain muscle mass. This means that the higher the male bodybuilders scored on controlled motivation for sport, the more they perceived benefits related to gain muscle mass and had the intention to gain muscle mass. Positive associations between controlled motivation and subjective norm have been observed when subjective norm is defined as social pressure to engage in future behaviors, therefore reflecting more controlling, externally-referenced beliefs about engaging in future health behaviors [3, 33]. The non-significant association between controlled motivation and perceived behavioral control might be explained by the observation that extrinsic regulation of motivation and perceived control are theoretically opposing constructs [24].

Autonomous and controlled motivations for sport were also expected to be, respectively, direct negative and positive predictors of DM. The results indicated that autonomous motivation for sport was non-significantly directly related to DM, but that controlled motivation for sport was significantly, directly and positively related to DM. This association was in agreement with the findings of Edwards et al. (2016) [22] who have associated perceived sociocultural pressure and controlled motivation according to their similarities, and have shown that perceived sociocultural pressure promoted the development of DM. Moreover, a recent study showed that individuals with needs and relatedness frustrations might be more oriented toward extrinsic goals such as achieving the perfect body or a hyper-muscular body, which could lead to maladaptive compensatory behaviors such as strict workouts and diets [23]. More specifically, Selvi and Bozo (2019) [23] showed that bodybuilders with high needs frustration had high scores of DM and muscle dysmorphia. The link between controlled motivation and DM was underlined in previous studies and our results confirmed it according to similar constructs. More generally, controlled motivation seems to be related to deviant behaviors, which is consistent with previous studies reporting that controlled motivation may be a risk factor for health behaviors and specifically doping-related variables [33, 59].

The examination of the mediating roles of the socio-cognitive variables from the theory of planned behavior (i.e., attitude, subjective norm, perceived behavioral control, and intention to gain muscle mass) in the relationship between motivations for sport and DM, helped us to shed light on the underlying motivational dynamics of DM. Autonomous motivation for sport was significantly, indirectly and positively related to DM through the mediating role of perceived behavioral control and intention to gain muscle mass. This means that the more male bodybuilders were self-determined motivated, the more they perceived that they controlled their behaviors to gain muscle mass and had the intention to gain muscle mass, and the more they were engaged in DM. Moreover, men who associate resistance training with masculinity were found to be less autonomously motivated for resistance training [60]. This result provides support to previous findings reporting that autonomous motivation was associated with gym attendance [60]. The results further showed that controlled motivation for sport was significantly, indirectly and positively related to DM via attitude and intention to gain muscle mass, and it was more strongly associated with DM than autonomous motivation for sport. According to previous research [29], the mediation results provide evidence that the regression scores of the indirect associations between the types of motivation for sport and DM were stronger than the regression scores of the direct associations. Effectively, autonomous motivation for sport was non-significantly related to DM directly, while it was significantly related indirectly. Moreover, the direct effect of controlled motivation for sport on DM was small compared to the indirect effect of DM via the variables of the theory of planned behavior. The pattern of effects provides evidence for another key mechanism in the model; controlled motivation for sport predicts future intentions and DM through the salient factors related to decision making, namely, attitude and intention to gain muscle mass. These results thus indicate that both autonomous and controlled motivations for sport contributed positively to DM via some of the theory of planned behavior variables, suggesting that, in line with the TCM predictions a complex motivational dynamic underlies DM in male bodybuilders.

Our study corroborates prior research showing that self-determination theory and the theory of planned behavior are complementary, as we demonstrated strong relationships between their constructs. The major contribution of this study is that the TCM, as a model integrating these two theories, offered a more complete explanation of the relationship between motivation for sport and DM in bodybuilders. However, the results differ somewhat from the pattern of effects found in previous tests of the TCM in other contexts, specifically regarding the role of self-determined motivation for sport. Given that DM is generally associated with detrimental health consequences [1], it might be hypothesized that DM is bivalent, sometimes resulting in deviant or at-risk behaviors and sometimes in healthy behavior. Another strength of this study is that the TCM was applied to a specific behavior (i.e., DM) in a specific context (i.e., bodybuilding).

Although this study has several strengths, a number of limitations should be acknowledged. First, our data are limited because our sample was composed only of young men with different levels of expertise (i.e., non-competitors, future competitors, competitors), which might have influenced their motivation and engagement in DM [61]. A possible solution would be to measure the level of competition and include it in the analyses. Moreover, these bodybuilders trained between 3 and 24 h per week, and the amount of practice time might have limited the homogeneity of our sample. Also, the sex orientation was not controlled among the male bodybuilders. Second, subjective norm is a questionable variable as it might indicate a measurement issue in the sense that such variables reflect greater pressure and controlling rather than supportive forms of motivation. They therefore are not likely to capture the shared variance between the need for relatedness and physical activity behaviors [27]. Importantly, the relationship between autonomous motivation and subjective norm casts doubt on the TCM, as the results to date have been varied, with studies from different countries finding positive, negative, or no relationships [37, 57, 62, 63]. Third, the data were collected using a self-report survey and might have been influenced by social desirability. Fourth, the internal consistency of some measures was quite low and this might have been linked to our heterogeneous sample or to the psychometric quality concerning the controlled motivation scale, or the inconsistency between the external regulation and the introjected regulation. Moreover, our study was correlational and had the typical limitations of this type of design; it would thus be interesting to conduct a longitudinal study to more closely examine the causal relationships between variables. The motivational mechanisms underlying DM could be fruitfully deepened by complementary analyses of motivational profiles. These future investigations would help to develop programs to equip bodybuilders to avoid the risks associated with DM. Methodologically, an important perspective for the future would be to determine a *cutoff* for DM, which might then function as a reference when using DM as a screening instrument for disordered eating. Moreover, eating disorders are very common in male bodybuilders [2] and it might be of value to examine the relationships between motivation for sport, DM and other specified feeding or eating disorders such as food restriction or night eating syndrome, or binge eating.

## Conclusions

Despite these limitations, the results indicate that strong relationships are embedded in the theoretically integrated model of self-determination theory and theory of planned behavior. This model is useful as it suggests the rationale behind the origins of the social cognitive variables of intention, attitude, and self-efficacy within the theory of planned behavior. The study showed that autonomous motivation for sport had a significant, indirect and positive association with DM via perceived behavioral control and intention to gain muscle, and that controlled motivation for sport was related to DM both directly and via attitude and intention to gain muscle mass. These results indicate that the TCM was partially supported in the context of bodybuilding, suggesting a complex motivational model underlying DM in male bodybuilders. Preventive actions may be important for male bodybuilders, who focus on gaining muscle mass. Specifically, such actions should be directed toward helping them to avoid developing controlled motivation because, although motivation for sport of any kind can be a direct or indirect risk factor for the development of deviant behavior, controlled motivation seems be put them at greater risk. It may therefore be important to carefully keep track of the development of motivation for sport to be sure that controlled motivation stays low, with a moderate and healthy practice of bodybuilding. Moreover, the motivational mechanisms explaining the development of DM could be deepened by complementary analyses in terms of motivational profiles.

## Data Availability

The datasets generated and/or analyzed during the current study are available from the corresponding author on reasonable request.

## Abbreviations

AIC: Akaike’s information criterion
AM: Autonomous motivation for sport
Att: Attitude to gain muscle mass
BREQ: Behavioral Regulation in Exercise Questionnaire
CFA: Confirmatory factor analysis
CFI: Comparative fit index
CM: Controlled motivation for sport
DM: Drive for muscularity behaviors
DMS: Drive for Muscularity Scale
DSM: Diagnostic and Statistical Manual of Mental Disorders
ECVI: Expected cross validation index
INT: Intention to gain muscle mass
MB: Muscularity behaviors
PBC: Perceived behavioral control related to gain muscle mass
PLOC: Perceived locus of causality
RMSEA: Root mean square error of approximation
SN: Subjective norm related to gain muscle mass
TCM: Rans-contextual model of motivation
TLI: Tucker-Lewis index
